# Supplementary material for: Evaluating the cost-effectiveness of polygenic risk score-stratified screening for abdominal aortic aneurysm
Source: Nat Commun. 2024 Sep 14;15:8063. doi: 10.1038/s41467-024-52452-w (PMC11401842; doi:10.1038/s41467-024-52452-w)
Supplement: Supplementary file 1 — Supplementary Information [file 41467_2024_52452_MOESM1_ESM.pdf]

Supplementary Information for "Evaluating the cost-effectiveness of polygenic risk score-stratified screening for abdominal aortic aneurysm"

Supplementary Methods

1 Effective sample size calculation

The effective sample size (N_{eff}) for a study of a binary trait is equivalent to the sample size as if in the same study the number of cases or controls would have been equal. If the number of cases and controls are known, the following formula may be used to obtain this quantity:

$$N_{eff} = \frac{4}{1/cases + 1/controls}.$$

In the scenario where the number of cases and controls is not known, N_{eff} may be imputed via the following formula[1]:

$$\widehat{N_{eff}} = \frac{4/\sigma^2{}_G - \beta^2}{\sigma_\beta{}^2},$$

where $\sigma^2{}_G$ is the variance of the genotypes, β is the log odds ratio of the effect allele and σ_β is the standard error of the log odds standard error. In the case of no genotype-level data being available, hence $\sigma^2{}_G$ is now known, it may be obtained from the allele frequencies of a matching reference panel via

$$\sigma^2{}_G = 2\,AF\ (1 - AF),$$

where AF is the allele frequency of the effect allele.

The effective sample size calculation becomes more complex in meta-analyses, as in that case different SNPs may have different numbers of cases/controls that were available at that particular locus, which then results in the sample size becoming a distribution. Analyses that involve weighted meta-analyses of genetically overlapping traits, the sample size calculation becomes even more difficult, as in that scenario we have to account for the fact that at each variant the aetiology may only be partially shared between the different traits. The formula in this scenario to obtain the total per-SNP effective sample size (N_{total}) becomes[2]:

$$N_{total} = N_{primary}\,(1 - \pi) + N_{meta}\pi\,(1 - \rho),$$

where $N_{primary}$ is the effective sample size of the target trait and N_{meta} is the effective sample size of the naive fixed-effects meta-analysis between the primary and adjunct traits. π is the local FDR (lFDR) providing the evidence of heterogeneity between the primary and adjunct trait at each SNP, and ρ is the overlap between the primary and adjunct studies.

Furthermore, the vast majority of SNPs are not expected to contribute to either trait, hence those SNP's N_{total} is going to be ~N_{meta}, as their lFDR will be ~1. Therefore, naively interpreting the summary association results from such weighted meta-analysis may lead to a greatly overestimated N_{total}, because even in unrelated traits a meta-analysis of null SNPs will yield the full combined sample size. Therefore, a more conservative estimate of N_{total} may be obtained by restricting the sample size calculation to only highly associated SNPs. By considering only the 447 SNPs with a combined association $p < 5 * 10^{-16}$, the average sample size in our study was thus found to be 312,458, in contrast to the naive estimate of 458,939, if all SNPs were considered.

2 Genome-wide association studies of the AAA and AAA-related phenotypes

Only HapMap3 panel of SNPs were considered, which were then filtered to eliminate those variants that failed QC in any of the UKB batches, or had a minor allele count < 20, or had an imputation INFO < 0.3. Samples were filtered out if they were not in the *"white.British.ancestry"* subset, or were too closely related, or were identified as sex discordant, all as defined in the UKB documentation. Finally, samples were also removed if they were part of the MetaGRS[3] study.

The UKB cohort was split into two non-overlapping subsets, one for model training and the other for testing. The training set included all individuals on the BILIVE study and the Interim release of the datasets to avoid the known biases arising to the selection of participants BiLEVE study[4], and to avoid any overlaps between the Nelson et al summary data[5] and our own test set. An additional 10,125 controls were removed from our analyses that were used by our collaborators at the University of Leicester for their own association study.

For the AAA GWAS we excluded individuals from the controls who were on anti-hypertensive or lipid lowering medications as well as anyone who was in the AAA Related case list. This process yielded 1,068 cases and 127,011 controls and 133,900 cases and 127,011 controls for the AAA and AAA Related association studies, respectively. In turn, our AAA test set consisted of 869 cases and 91,012 controls, including 730 incident cases up until the age of 80 (740 in total).

For the genetic association step in our training set, we defined cases as those individuals who manifested the condition at any time within our records (as either prevalent or incident cases). For the model fit we also added in the following covariates: *age*, *sex*, *batch*chip* and the first ten principal components of ancestry. Association between phenotype and genotype was performed via PLINK2's[6] '*--glm firth-fallback*' function.

3 Processing and quality control steps of association summary data

The Malik et al[7] and the AAAGen summary data lacked the per SNP breakdown of cases/controls, thus the effective sample size was imputed by the previously described method. Variants from all summary data were filtered to remove ambiguous SNPs (A/T and G/C), and those markers that were found to violate the following quality-control thresholds:

$$\sigma_{SS} < 0.5 \ \sigma_G \ or \ \sigma_{SS} > \sigma_G + 0.1 \ or \ \sigma_{SS} < 0.1 \ or \ \sigma_G < 0.05 ,$$

where σ_{SS} is defined as

$$\sigma_{SS} \quad = \frac{2}{\sqrt{N_{eff} \quad \sigma_{\beta}{}^{2}}}.$$

The above threshold values used for this filtering step were sourced from the LDpred2 recommended settings[8].

Information from traits with overlapping genetic aetiologies may be combined to increase predictive performance of a PRS[2]. Therefore, to exploit this genetic correlation, we combined summary data via shaPRS in the following sequence, AAAGen, AAA in UKB, AAA Related, CAD and stroke. Note, we found that starting model training from the larger, better powered AAAGen study provided higher final accuracy results, as opposed to starting the AAA UKB study. We verified that the addition of each new summary data improved results at each stage. We also compared our results against combining all summary data in a single step via MTAG[9], but found that combining the summary data via shaPRS performed better (an AUC of 0.699 vs 0.706, for MTAG vs shaPRS, respectively). We performed formal tests of significance on all PRS' via r2redux's r2_var function comparing the model against the null, and also comparing each PRS against the best model via pROC' Delong' tests. We evaluated our models for miscalibration via a Hosmer-Lemeshow test modified for larger sample sizes[10], which, with the exception of All AAA + AAA-related + CAD + stroke via MTAG+PRS-CS (0.00422), did not find evidence for PRS miscalibration. Finally, we also compared PRS-CS against LDpred2 at fitting the best model, and found that LDpred2 provided a superior performance with an AUC of 0.707 vs 0.708 for PRS-CS vs LDpred2, respectively.

We ensured that our Test set evaluations were free from overfitting by working with a version of the AAAGen data that excluded the UKB. The full details of all PRS mode evaluation results can be found in Supplementary Table 3.

4 Sensitivity analyses exploring impact of missing data

We summarised missingness and explored the impact on results through the use of multiple imputation (MI) in a sensitivity analysis. Missing values were generated for risk factors using chained equations to create 20 imputed datasets with 10 burn-ins. Binary variables (alcohol intake, family history of CVD, diabetes, anti-hypertensive drugs, lipid-lowering drugs) were imputed using logistic regression, and smoking status using multinomial logit. Continuous variables (BMI, SBP, total cholesterol, HDL cholesterol) were imputed using predictive mean matching. The outcome indicator and cumulative hazard function (estimated with the Nelson-Aalen estimator) were included in all imputation models. Results from the 20 imputations were combined using Rubin's rules to provide final estimates and are provided in Supplementary Table 6.

Supplementary Table 1 | AAA study details		
**Phenotype**	**N cases**	**N Controls**
UKB AAA	1,068	127,011
UKB AAA-related	133,900	127,011
AAAGen	~104,179.4	

The number for cases/controls specified is the maximum per each study. Note, for the AAAGen dataset the number of cases/controls was unavailable, therefore the per-SNP effective sample size was imputed by the previously described methods. Thus the number quoted in the table is the median effective sample size across all SNPs. 'N Cases' refers to AAA cases for the UKB AAA and AAAGen datasets, and to a a composite phenotype of conditions potentially genetically overlapping with AAA identified from the literature for the UKB AAA-related dataset.

Supplementary Table 2 | UK Biobank Phenotype definitions used in PRS development	
**Phenotype**	**ICD10 and OPSC4 codes**
AAA	**fatal_icd10:** I71.3 (Abdominal aortic aneurysm, ruptured), I71.4 (Abdominal aortic aneurysm, without rupture)<br>**nonfatal_icd10:** I71.3 (Abdominal aortic aneurysm, ruptured), I71.4 (Abdominal aortic aneurysm, without rupture)<br>**opcs4:** L18.3 (Emergency suprarenal open repair of AAA), L18.4 (Emergency infra-renal open repair of AAA), L18.5 (Emergency open repair of AAA (other)), L18.6 (Emergency infra-renal open repair of AAA (bifurcated)), L19.3 (Elective suprarenal open repair of AAA), L19.4 (Elective infra-renal open repair of AAA (straight graft)), L19.5 (Elective infra-renal open repair of AAA (other)), L19.6 (Elective infra-renal open repair of AAA (bifurcated graft), L27.1 (Infra-renal EVAR), L27.2 (Suprarenal EVAR), L27.5 (EVAR at bifurcation NEC), L27.6 (Monoiliac EVAR), L28.1 (Infra-renal EVAR), L28.5 (EVAR at bifurcation NEC), L28.6 (Monoiliac EVAR)

AAA-related	**fatal_icd10:** Q87.4 (Marfan's syndrome), I21 (Acute myocardial infarction), I23 (Certain current complications following ST elevation (STEMI) and non-ST elevation (NSTEMI) myocardial infarction (within the 28 day period)), I22 (Subsequent ST elevation (STEMI) and non-ST elevation (NSTEMI) myocardial infarction), I25.2 (Old myocardial infarction), I70.2 (Atherosclerosis of native arteries of extremities with gangrene), I83 (Varicose veins of lower extremities), I86 (Varicose veins of other sites), Q79.6 (Ehlers-Danlos syndromes), M35.2 (Behçet's disease), M02.3 (Reiter's disease), M08.1 (Juvenile ankylosing spondylitis), M45 (Ankylosing spondylitis), E11 (Type 2 diabetes mellitus), M31.5 (Giant cell arteritis with polymyalgia rheumatica), M31.6 (Other giant cell arteritis), Q25.1 (Coarctation of aorta), Q61 (Cystic kidney disease), Q44.6 (Cystic disease of liver), E78 (Disorders of lipoprotein metabolism and other lipidemias), I10 (Essential (primary) hypertension)<br>**nonfatal_icd10:** same codes as fatal_icd10

AAA phenotype is aortic abdominal aneurysm and AAA-related is a composite phenotype of conditions potentially genetically overlapping with AAA identified from the literature.

Supplementary Table 3 | PRS model performance in test set

**Training dataset PRS is derived on**	**combined via**	**PRS**	**r^2 (SD)**	**AUC (LB -UB)**	**p**	**HL p**	**diff to best**
UKB AAA	N/A	PRS-CS	0.00034 ($1.480*10^{-5}$)	0.551 (0.532 - 0.570)	$2.92 *10^{-8}$	0.546	$1.83 *10^{-42}$
AAAGen			0.00405 ($1.480*10^{-5}$)	0.682 (0.665 - 0.700)	$1.47* 10^{-83}$	0.703	$5.99 *10^{-5}$
All AAA (starting from UKB)	MTAG		0.00444 ($1.65*10^{-5}$)	0.692 (0.675 - 0.709)	$2.02* 10^{-91}$	0.136	0.00762
	shaPRS		0.00432 ($1.65*10^{-5}$)	0.689 (0.672 - 0.706)	$4.12* 10^{-89}$	0.417	0.00128
All AAA (starting from AAAGen)	MTAG		0.00317 ($1.77*10^{-5}$)	0.665 (0.647 - 0.683)	$9.48* 10^{-66}$	0.712	$4.28 *10^{-9}$

	shaPRS		0.00414 (1.71*10^{-5})	0.684 (0.667 - 0.701)	1.99 * 10^{-85}	0.548	0.000131
All AAA + AAA-related	MTAG		0.00442 (1.39 *10^{-5})	0.691 (0.674 - 0.708)	4.32*10^{-91}	0.404	0.00302
	shaPRS		0.00507 (1.53*10^{-5})	0.704 (0.687 - 0.721)	2.42 * 10^{-104}	0.473	0.364
All AAA + AAA-related + CAD	**shaPRS**		0.00530 (1.66*10^{-5})	0.707 (0.690 - 0.724)	5.12*10^{-109}	0.9246	0.733
		**LDpred2**	**0.00547 (1.52*10^{-5})**	**0.708 (0.691 - 0.725)**	**1.98*10^{-112}**	**0.565**	**N/A**
All AAA + AAA-related + stroke		PRS-CS	0.00516 (1.36*10^{-5})	0.705 (0.688 - 0.721)	2.98*10^{-106}	0.484	0.485
All AAA + AAA-related + CAD + stroke	MTAG		0.00497 (1.6*10^{-5})	0.699 (0.682 - 0.716)	3.15*10^{-102}	0.00422	0.0749
	shaPRS		0.00527 (1.53*10^{-5})	0.706 (0.689 - 0.723)	1.87*10^{-108}	0.986	0.683
		LDpred2	0.00542 (1.45*10^{-5})	0.707 (0.690 - 0.724)	1.33*10^{-111}	0.615	0.678
PGS003973 / PRSAAA_woUKB [11]	N/A	N/A	0.00444 (1.56*10^{-5})	0.693 (0.676 - 0.710)	1.89*10^{-91}	0.441	0.0193
PGS001784 / 1kgeur_gbmi_leaveUKBBout_AAA_pst_eff_a1_b0.5_phiauto [12]	N/A	PRS-CS	0.00134 (1.53*10^{-5})	0.608 (0.589 - 0.626)	1.25*10^{-28}	0.0621	1.02*10^{-21}

Results for each of the datasets, PRS models and methods evaluated. The **data** column lists the studies that were used in the construction of the PRS. The **combined via** column displays the methods that were used to combine the association information from the studies. The **PRS** column contains the PRS construction methods that were used to generate the final PRS. Both PRS-CS and LDpred2 generated their PRS via their respective 'auto' options. The $\mathbf{r^2}$ and **AUC** columns show the performance of the final PRS for each study calculated for differentiating AAA cases from controls in

the test set. The r^2 **SD** is calculated as the standard deviation of the squared correlation between the observed and predicted trait values resampled a 1,000 times. The AUC lower bound (**LB**) and upper bound (**UB**) represent the 95% confidence intervals which were computed with 2,000 stratified bootstrap replicates. Note, the AUC is calculated from a simple binary outcome including prevalent and incident events combined,with PRS included as a continuous variable with no other predictors included. The **p** column shows the 'r2redux' r2_var test's (one-sided) p-value, which formally evaluates the significance of the PRS. The **HL p** column shows the (one-sided) p-value from a Hosmer–Lemeshow test modified for large sample sizes[10]. **diff to best** column shows the pROC' Delong' test's (two-sided) p-value , which formally evaluates if there is a significant difference between a given PRS and the PRS with the highest performance (All AAA + AAA-related + CAD via shaPRS+LDpred2).

**Supplementary Table 4 | Incident AAA code list used for time-to-event modelling**	
ICD codes	I71.3 (Abdominal aortic aneurysm, ruptured)*<br>I71.4 (Abdominal aortic aneurysm, without rupture)*
OPCS codes	L18.3 (Emergency suprarenal open repair of AAA), L18.4 (Emergency infra-renal open repair of AAA), L18.5 (Emergency open repair of AAA (other)) ,L18.6 (Emergency infra-renal open repair of AAA (bifurcated))<br>L19.3 (Elective suprarenal open repair of AAA), L19.4 (Elective infra-renal open repair of AAA (straight graft)), L19.5 (Elective infra-renal open repair of AAA (other)),L19.6 (Elective infra-renal open repair of AAA (bifurcated graft))<br>L27.1 (Infra-renal EVAR), L27.2 (Suprarenal EVAR), L27.5 (EVAR at bifurcation NEC), L27.6 (Monoiliac EVAR)<br>L28.1 (Infra-renal EVAR), L28.2 (Suprarenal EVAR), L28.5 (EVAR at bifurcation NEC), L28.6 (Monoiliac EVAR)

Supplementary Table 5 | Summary of risk factors in UKB test set (N=91,731 with a PRS score)			
	**All**	**Males**	**Females**
N	91,731	38,425	53,306
Complete case N	72,928 (79.5%)	30,426 (79.2%)	42,502 (79.7%)
Age at entry*	56 (49, 62)	56 (49, 62)	57 (49, 62)
BMI (kg/m^2)**	26.6 (4.3)	27.1 (3.8)	26.2 (4.6)
Missing	202 (0.2%)	83 (0.2%)	119 (0.2%)
Townsend index*	-2.5 (-3.8, -0.2)	-2.5 (-3.8, -0.1)	-2.5 (-3.8, -0.3)
Missing	114 (0.1%)	46 (0.1%)	68 (0.1%)
Smoking status			
Never	37,038 (40%)	13,528 (35%)	23,510 (44%)
Ex	46,520 (51%)	20,695 (54%)	25,825 (49%)
Current	7,934 (9%)	4,101 (11%)	3833 (7%)
Missing	239 (0.3%)	101 (0.3%)	138 (0.3%)
Any alcohol	86,677 (95%)	36,842 (96%)	49,835 (93%)
Missing	55 (<0.1%)	23 (<0.1%)	32 (<0.1%)
SBP (mm Hg)**	134.9 (17.6)	138.4 (16.3)	132.4 (18.0)
Missing	55 (<0.1%)	11 (<0.1%)	44 (<0.1%)
Anti-hypertensive drugs	7,646 (8.4%)	3,875 (10.1%)	3,771 (7.1%)
Missing	603 (0.7%)	333 (0.9%)	270 (0.5%)
Diabetes	1,203 (1.3%)	752 (2.0%)	451 (0.9%)
Missing	122 (0.2%)	65 (0.2%)	57 (0.1%)
Family history of CVD	47,278 (56%)	18,511 (53%)	28,767 (58%)
Missing	6,845 (7.5%)	3,377 (8.8%)	3,468 (6.5%)
Total cholesterol (mmol/L)**	5.8 (1.1)	5.7 (1.0)	5.9 (1.1)
Missing	4156 (4.5%)	1698 (4.4%)	2458 (4.6%)
HDL cholesterol (mmol/L)**	1.5 (0.4)	1.3 (0.3)	1.6 (0.4)
Missing	11,763 (12.8%)	4,531 (11.8%)	7,232 (13.6%)
Lipid lowering drugs	7,292 (8.0%)	4,087 (10.7%)	3,205 (6.0%)
Missing	603 (0.7%)	333 (0.9%)	270 (0.5%)

* median (IQR)   ** mean (SD)

Note: percentages given of those non-missing for each risk factor

Supplementary Table 6 | Summary of observations by sub-group				
**PRS tertile**	**Smoking status**	**Observed N**	**Observed proportion of population**	**Observed number of AAA events (% of subgroup)***
**Men**				
All	All	30,246	100%	464 (1.53%)
Low	All	10,770	35.4%	58 (0.54%)
Intermediate		9,908	32.6%	136 (1.37%)
High		9,748	32.0%	270 (2.77%)
All	Never	10,902	35.8%	56 (0.51%)
	Ex	16,426	54.0%	276 (1.68%)
	Current	3,098	10.2%	132 (4.26%)
Low	Never	3,960	13.0%	16 (0.40%)
Intermediate		3,537	11.6%	17 (0.48%)
High		3,405	11.2%	23 (0.68%)
Low	Ex	5,850	19.2%	25 (0.43%)
Intermediate		5,365	17.6%	78 (1.45%)
High		5,211	17.1%	173 (3.32%)
Low	Current	960	3.2%	17 (1.77%)
Intermediate		1,006	3.3%	41 (4.08%)
High		1,132	3.7%	74 (6.54%)
**Women**				
All	All	42,502	100%	81 (0.19%)
Low	All	14,981	35.3%	14 (0.09%)
Intermediate		13,687	32.3%	22 (0.16%)
High		13,834	32.6%	45 (0.33%)
All	Never	18,941	44.6%	17 (0.09%)
	Ex	20,652	48.6%	41 (0.20%)
	Current	2,909	6.9%	23 (0.79%)
Low	Never	6,681	15.7%	4 (0.06%)
Intermediate		6,254	14.7%	4 (0.06%)
High		6,006	14.1%	9 (0.15%)
Low	Ex	7,433	17.5%	5 (0.07%)
Intermediate		6,503	15.3%	11 (0.17%)
High		6,716	15.8%	25 (0.37%)
Low	Current	867	2.0%	5 (0.58%)
Intermediate		930	2.2%	7 (0.75%)
High		1,112	2.6%	11 (0.99%)

* observed during whole follow-up period

**Supplementary Table 7 | Hazard ratios for recorded AAA from Cox regression, with multiply imputed dataset (N = 91,731)**		
**Risk factor**	**HR (95% CI)**	**p-value***
PRS group		
Low risk	1	
Intermediate risk	2.33 (1.81, 3.01)	<0.001
High risk	4.46 (3.52, 5.66)	
Sex		
Female	1	<0.001
Male	4.77 (3.82, 5.95)	
Townsend deprivation index (per 1 unit increase)	1.02 (1.00, 1.05)	0.05
Alcohol intake		
Non-drinker	1	<0.001
Drinker	0.56 (0.43, 0.72)	
Family history of CVD		
No	1	0.6
Yes	1.04 (0.88, 1.22)	
Diabetic		
No	1	0.09
Yes	1.26 (0.96, 1.65)	
Smoking status		
Never smoker	1	
Ex-smoker	2.39 (1.90, 2.99)	<0.001
Current smoker	8.07 (6.31, 10.33)	
BMI (per kg/m^2 increase)	1.01 (0.99, 1.03)	0.5
Systolic blood pressure (per 10mm Hg)	0.98 (0.94, 1.03)	0.4
Anti-hypertensive medication		<0.001
No	1	
Yes	2.83 (2.38, 3.37)	
Total cholesterol (per mmol/L)	1.11 (1.03, 1.21)	0.01
HDL cholesterol (per mmol/L)	0.28 (0.20, 0.38)	<0.001
Lipid-lowering medication		<0.001
No	1	
Yes	2.73 (2.26, 3.29)	

* two-sided Wald test without correction for multiple comparisons

Supplementary Figure 1 Study Design.

PRS = polygenic risk score; UKB = UK Biobank; GWAS = genome-wide association study; CAD = coronary artery disease; AAA = abdominal aortic aneurysm.

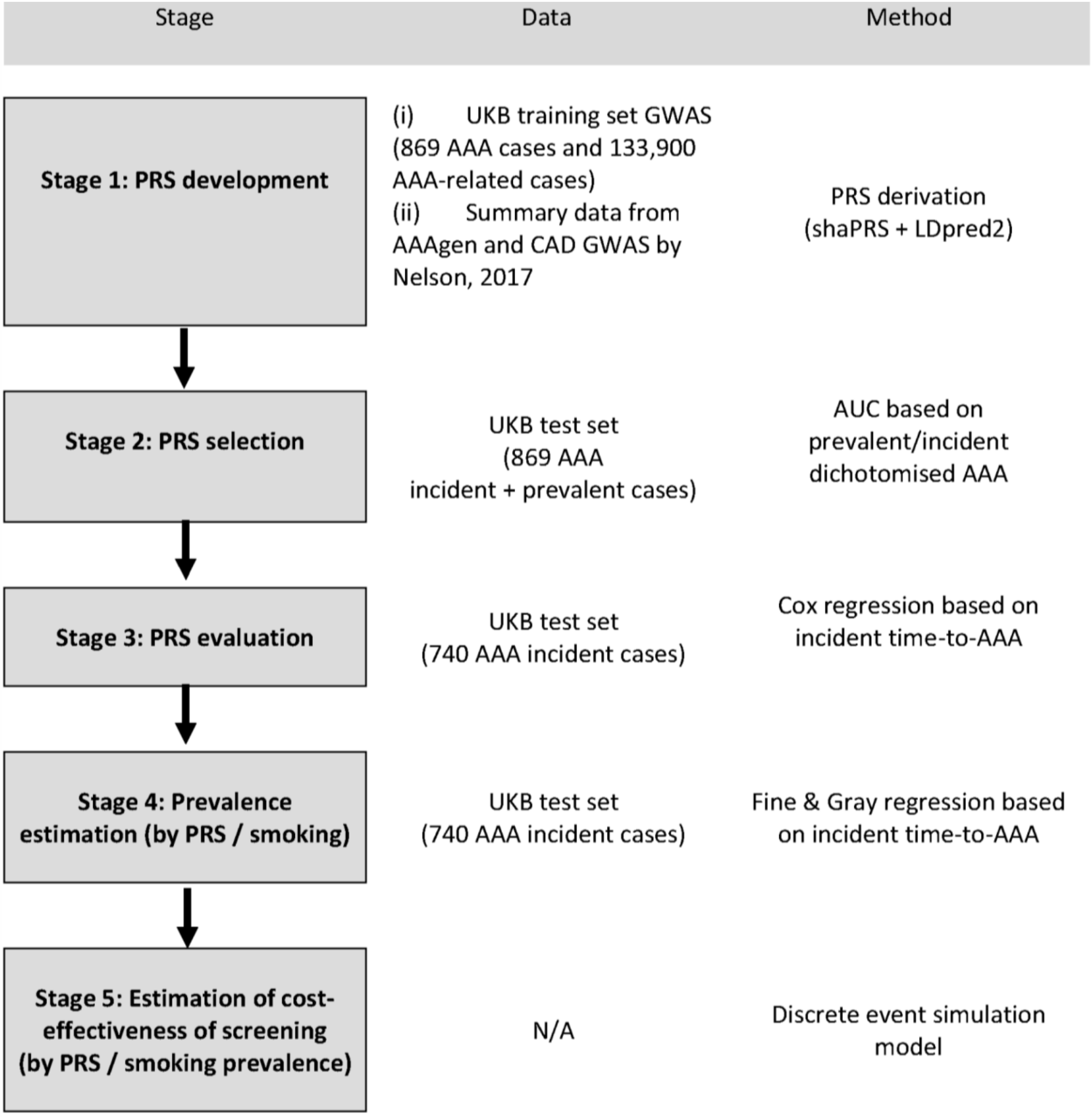

Supplementary Figure 2 Incremental net benefit compared to no invitation, by age at invitation and baseline prevalence at age 60 in men.

INB is evaluated at a willingness-to-pay of £30,000 per QALY, based on 1M hypothetical individuals in the DES. Points plotted are point estimates with 95% uncertainty interval derived from 100 bootstrap PSA samples. Separate PRS and smoking sub-group prevalences estimated from UKB test set as CIF x inflation factor; indicated on the x-axis (PRS1 = low PRS risk group, PRS2 = intermediate PRS risk group, PRS3 = high PRS risk group; never = never smoker, ex = ex-smoker, curr = current smoker). INB = incremental net benefit; QALY = quality-adjusted life-year; DES = discrete event simulation; PSA = probabilistic sensitivity analysis; PRS = polygenic risk score; UKB = UK Biobank; CIF = cumulative incidence function; AAA = abdominal aortic aneurysm.

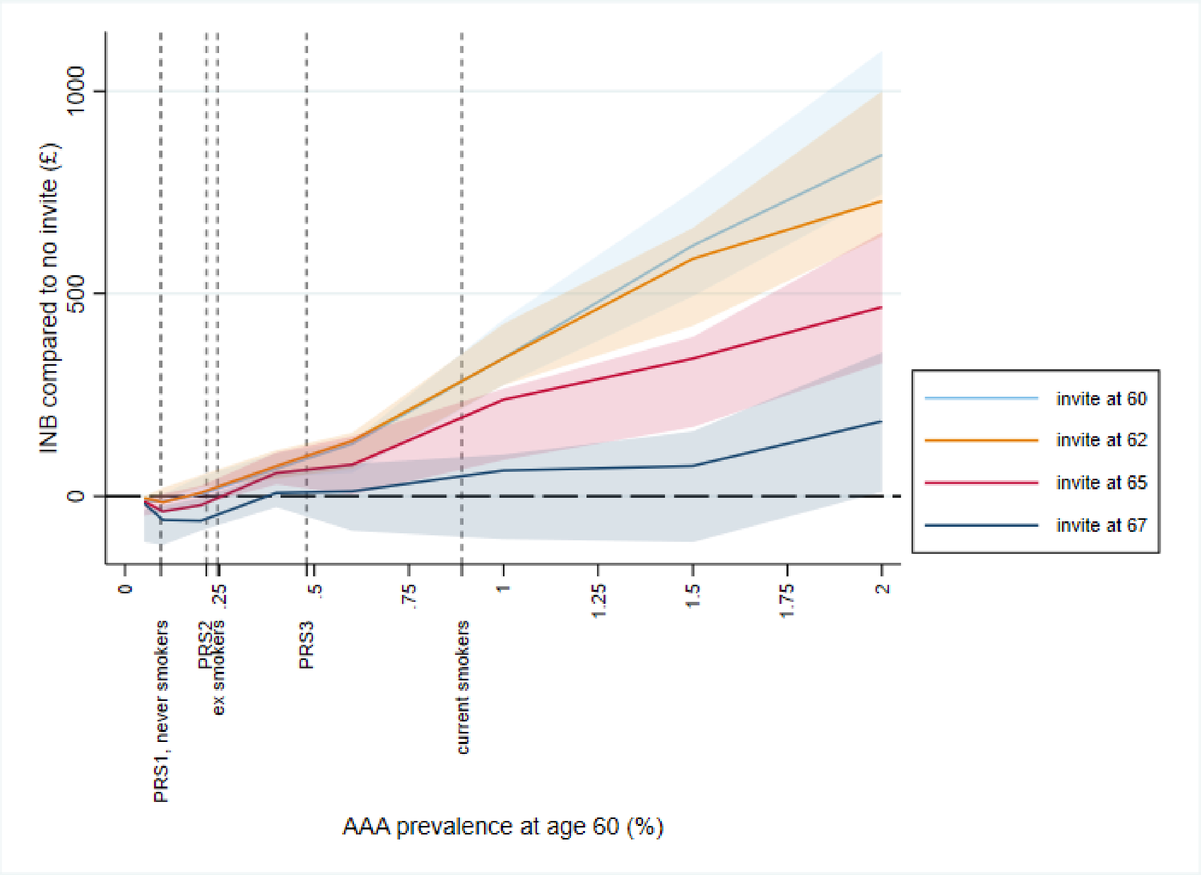

Supplementary Figure 3 Incremental net benefit compared to no invitation, by age at invitation and baseline prevalence at age 65 in women.

INB is evaluated at a willingness-to-pay of £30,000 per QALY, based on 1M hypothetical individuals in the DES. Points plotted are point estimates with 95% uncertainty interval derived from 100 bootstrap PSA samples. Separate PRS and smoking sub-group prevalences estimated from UKB test set as CIF x inflation factor; indicated on the x-axis (PRS1 = low PRS risk group, PRS2 = intermediate PRS risk group, PRS3 = high PRS risk group; never = never smoker, ex = ex-smoker, curr = current smoker). INB = incremental net benefit; QALY = quality-adjusted life-year; DES = discrete event simulation; PSA = probabilistic sensitivity analysis; PRS = polygenic risk score; UKB = UK Biobank; CIF = cumulative incidence function; AAA = abdominal aortic aneurysm.

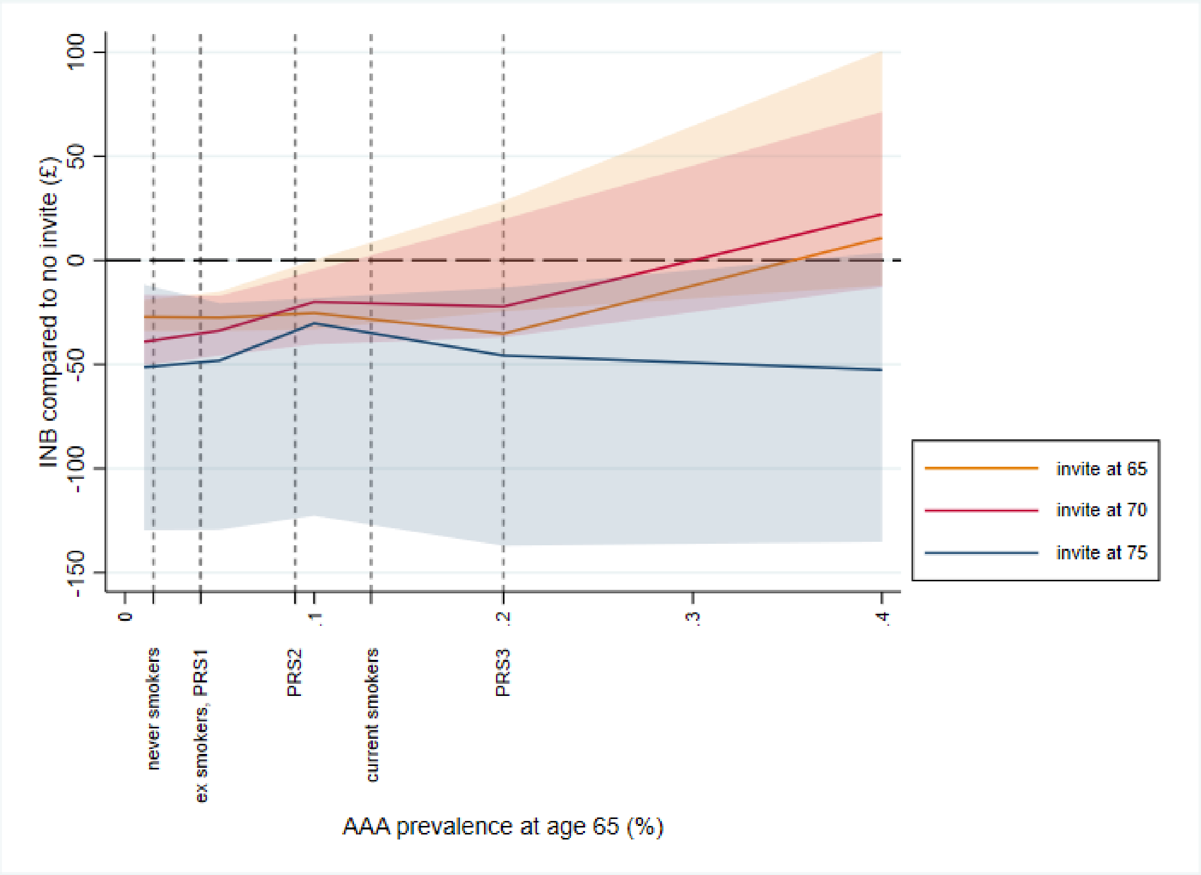

Supplementary Figure 4 Incremental net benefit compared to no invitation, by age at invitation and baseline prevalence at age 60 in men.

INB is evaluated at a willingness-to-pay of £20,000 per QALY, based on 1M hypothetical individuals in the DES. Points plotted are point estimates with 95% uncertainty interval derived from 100 bootstrap PSA samples. PRS/smoking sub-group prevalences estimated from UKB test set as CIF x inflation factor; indicated on the x-axis (PRS1 = low PRS risk group, PRS2 = intermediate PRS risk group, PRS3 = high PRS risk group; never = never smoker, ex = ex-smoker, curr = current smoker). INB = incremental net benefit; QALY = quality-adjusted life-year; DES = discrete event simualtion; PSA = probabilistic sensitivity analysis; PRS = polygenic risk score; UKB = UK Biobank; CIF = cumulative incidence function; AAA = abdominal aortic aneurysm.

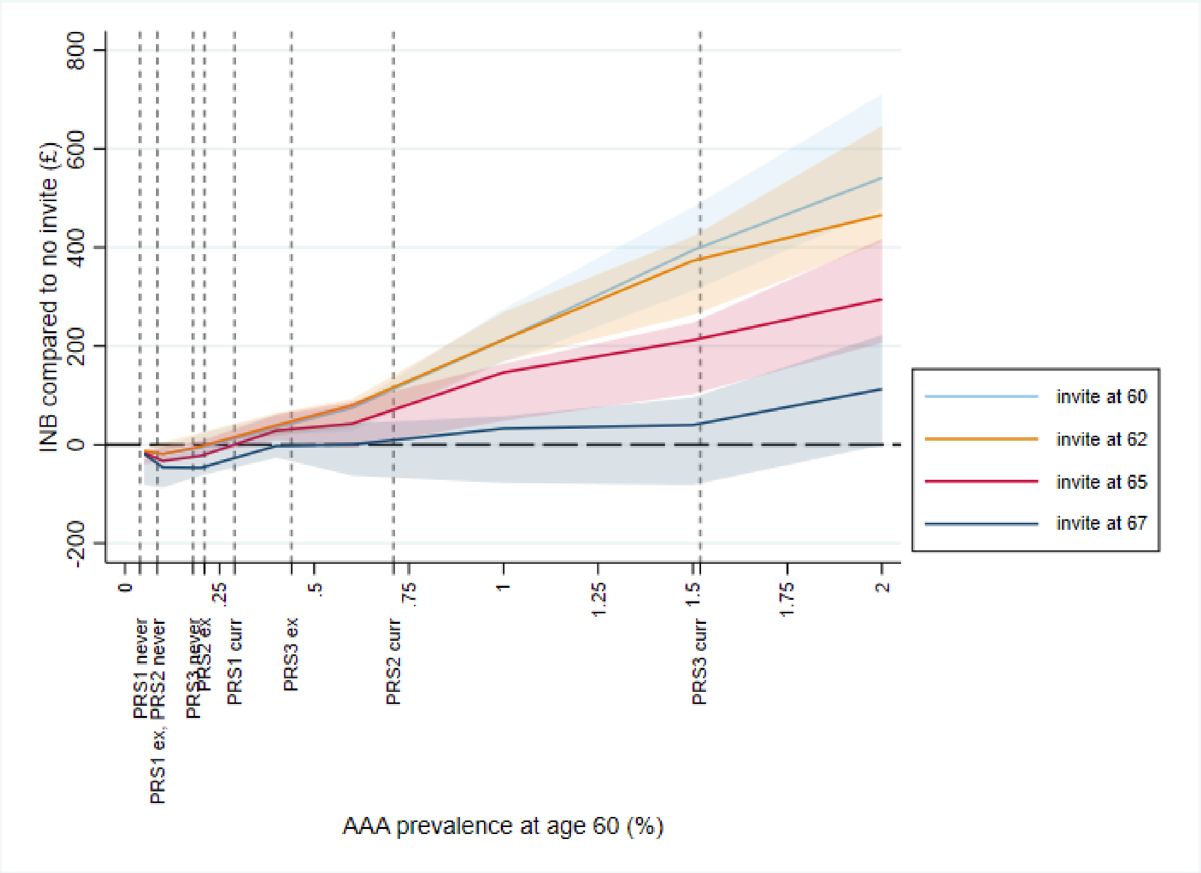

Supplementary Figure 5 Incremental net benefit compared to no invitation, by age at invitation and baseline prevalence at age 65 in women.

INB is evaluated at a willingness-to-pay of £20,000 per QALY, based on 1M hypothetical individuals in the DES. Points plotted are point estimates with 95% uncertainty interval derived from 100 bootstrap PSA samples. PRS/smoking sub-group prevalences estimated from UKB test set as CIF x inflation factor; indicated on the x-axis (PRS1 = low PRS risk group, PRS2 = intermediate PRS risk group, PRS3 = high PRS risk group; never = never smoker, ex = ex-smoker, curr = current smoker). INB = incremental net benefit; QALY = quality-adjusted life-year; DES = discrete event simulation; PSA = probabilistic sensitivity analysis; PRS = polygenic risk score; UKB = UK Biobank; CIF = cumulative incidence function; AAA = abdominal aortic aneurysm.

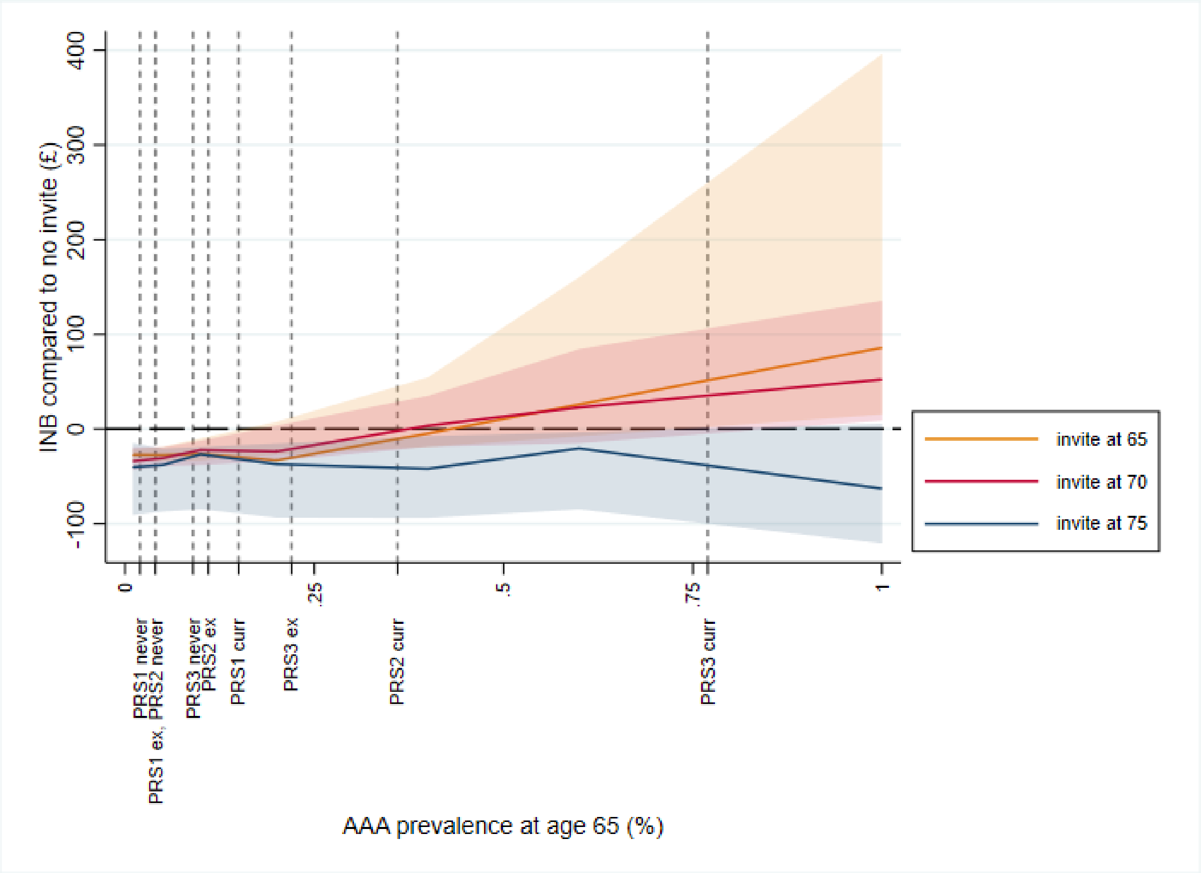

Supplementary References

1. Privé, F., Arbel, J., Aschard, H. & Vilhjálmsson, B. J. Identifying and correcting for misspecifications in GWAS summary statistics and polygenic scores. *bioRxiv* 2021.03.29.437510 (2022) doi:10.1101/2021.03.29.437510.
2. Kelemen, M., Vigorito, E., Anderson, C. A. & Wallace, C. ShaPRS: Leveraging shared genetic effects across traits or ancestries improves accuracy of polygenic scores. *bioRxiv* (2021) doi:10.1101/2021.12.10.21267272.
3. Abraham, G. *et al.* Genomic risk score offers predictive performance comparable to clinical risk factors for ischaemic stroke. *Nat. Commun.* **10**, 5819 (2019).
4. Miller, S. *et al.* The UK BiLEVE Study: The First Genetic Study in UK Biobank Identifies Novel Regions Associated with Airway Obstruction and Smoking Behaviour. in *B31. INFLAMMATION AND COPD* A2714–A2714 (American Thoracic Society, 2015).
5. Nelson, C. P. *et al.* Association analyses based on false discovery rate implicate new loci for coronary artery disease. *Nat. Genet.* **49**, 1385–1391 (2017).
6. Chang, C. C. *et al.* Second-generation PLINK: rising to the challenge of larger and richer datasets. *Gigascience* **4**, 7 (2015).
7. Malik, R. *et al.* Multiancestry genome-wide association study of 520,000 subjects identifies 32 loci associated with stroke and stroke subtypes. *Nat. Genet.* **50**, 524–537 (2018).
8. Privé, F., Arbel, J. & Vilhjálmsson, B. J. LDpred2: better, faster, stronger. *Bioinformatics* **36**, 5424–5431 (2020).
9. Turley, P. *et al.* Multi-trait analysis of genome-wide association summary statistics using MTAG. *Nat. Genet.* **50**, 229–237 (2018).
10. Nattino, G., Pennell, M. L. & Lemeshow, S. Assessing the goodness of fit of logistic regression models in large samples: A modification of the Hosmer-Lemeshow test. *Biometrics* **76**, (2020).
11. Roychowdhury, T. *et al.* Genome-wide association meta-analysis identifies risk loci for abdominal aortic aneurysm and highlights PCSK9 as a therapeutic target. *Nat. Genet.* **55**, (2023).

12. Global Biobank analyses provide lessons for developing polygenic risk scores across diverse cohorts. *Cell Genomics* **3**, 100241 (2023).
