# Supplementary material for: Evaluating the cost-effectiveness of polygenic risk score-stratified screening for abdominal aortic aneurysm
Source: Nat Commun. 2024 Sep 14;15:8063. doi: 10.1038/s41467-024-52452-w (PMC11401842; doi:10.1038/s41467-024-52452-w)
Supplement: Supplementary file 3 — Reporting Summary [file 41467_2024_52452_MOESM3_ESM.pdf]

nature portfolio

Corresponding author(s): LG Kim

Last updated by author(s): Aug 19, 2024

Reporting Summary

Nature Portfolio wishes to improve the reproducibility of the work that we publish. This form provides structure for consistency and transparency in reporting. For further information on Nature Portfolio policies, see our Editorial Policies and the Editorial Policy Checklist.

Statistics

For all statistical analyses, confirm that the following items are present in the figure legend, table legend, main text, or Methods section.

n/a	Confirmed	
☐	☒	The exact sample size (*n*) for each experimental group/condition, given as a discrete number and unit of measurement
☐	☒	A statement on whether measurements were taken from distinct samples or whether the same sample was measured repeatedly
☐	☒	The statistical test(s) used AND whether they are one- or two-sided<br>*Only common tests should be described solely by name; describe more complex techniques in the Methods section.*
☐	☒	A description of all covariates tested
☐	☒	A description of any assumptions or corrections, such as tests of normality and adjustment for multiple comparisons
☐	☒	A full description of the statistical parameters including central tendency (e.g. means) or other basic estimates (e.g. regression coefficient) AND variation (e.g. standard deviation) or associated estimates of uncertainty (e.g. confidence intervals)
☐	☒	For null hypothesis testing, the test statistic (e.g. *F*, *t*, *r*) with confidence intervals, effect sizes, degrees of freedom and *P* value noted<br>*Give P values as exact values whenever suitable.*
☒	☐	For Bayesian analysis, information on the choice of priors and Markov chain Monte Carlo settings
☒	☐	For hierarchical and complex designs, identification of the appropriate level for tests and full reporting of outcomes
☐	☒	Estimates of effect sizes (e.g. Cohen's *d*, Pearson's *r*), indicating how they were calculated

Our web collection on statistics for biologists contains articles on many of the points above.

Software and code

Policy information about availability of computer code

Data collection	No new data were collected for this study, i.e. there is no relevant software/code relating to data collection.
Data analysis	Code to perform all PRS development analyses reported in this manuscript is available at github.com/mkelcb/aaa-paper. The final AAA PRS file is available from the Supplementary data and from the PGS Catalog with score ID PGS003429. All post-PRS-development statistical analyses are performed using Stata v17.0. DES modelling is conducted using R v4.2.1. The full DES model is available at https://github.com/mikesweeting/AAA_DES_model.

For manuscripts utilizing custom algorithms or software that are central to the research but not yet described in published literature, software must be made available to editors and reviewers. We strongly encourage code deposition in a community repository (e.g. GitHub). See the Nature Portfolio guidelines for submitting code & software for further information.

Data

Policy information about availability of data

All manuscripts must include a data availability statement. This statement should provide the following information, where applicable:

- Accession codes, unique identifiers, or web links for publicly available datasets
- A description of any restrictions on data availability
- For clinical datasets or third party data, please ensure that the statement adheres to our policy

The best performing AAA PRS file generated in this study has been deposited in the PGS Catalog database under accession code PGS003429 (https://www.pgscatalog.org/score/PGS003429/). This research has been conducted using the UK Biobank Resource under Application Number 7439 (https://www.ukbiobank.ac.uk). Copyright © 2023, NHS England. Re-used with the permission of the NHS England [and/or UK Biobank]. All rights reserved. This research used data assets made available by National Safe Haven as part of the Data and Connectivity National Core Study, led by Health Data Research UK in partnership with the Office for National Statistics and funded by UK Research and Innovation (research which commenced between 1st October 2020 – 31st March 2021 grant ref MC_PC_20029; 1st April 2021 -30th September 2022 grant ref MC_PC_20058). For the purpose of open access, the author has applied a Creative Commons Attribution (CC BY) licence to any Author Accepted Manuscript version arising from this submission.

Research involving human participants, their data, or biological material

Policy information about studies with human participants or human data. See also policy information about sex, gender (identity/presentation), and sexual orientation and race, ethnicity and racism.

Reporting on sex and gender	Only sex was collected in these studies, and has been reported as such.
Reporting on race, ethnicity, or other socially relevant groupings	The Townsend deprivation index is a standard measure of social deprivation and was available as part of the publicly available data we used. Adjustment of relevant analyses for confounders is described in the text.
Population characteristics	This is provided for the UKB test set in Suppl Table S5.
Recruitment	Participants were not recruited as part of this modelling study. The potential impact of selection bias into the data sources used is described in the manuscript.
Ethics oversight	This is a modelling study.

Note that full information on the approval of the study protocol must also be provided in the manuscript.

Field-specific reporting

Please select the one below that is the best fit for your research. If you are not sure, read the appropriate sections before making your selection.

☒ Life sciences ☐ Behavioural & social sciences ☐ Ecological, evolutionary & environmental sciences

For a reference copy of the document with all sections, see nature.com/documents/nr-reporting-summary-flat.pdf

Life sciences study design

All studies must disclose on these points even when the disclosure is negative.

Sample size	Details for effective sample size for PRS are provided in the online methods. DES results are based on 1M hypothetical individuals to obtain a balance between computational run time and the need to obtain reliable estimates of cost-effectiveness measures and numbers of events.
Data exclusions	There are no exclusions other than removal of AAAGen data from UKB to prevent overlap between datasets used for analysis; this is explained in the online methods and numbers used are provided.
Replication	In the DES, the random seed is set to a known value to ensure results are reproducible. atasets for the PRS development were separated into training and testing sets to improve generalization.
Randomization	The data used are observational. Relevant results are adjusted for confounding (eg Table 1; see online methods).
Blinding	There is no group allocation in this study.

Reporting for specific materials, systems and methods

We require information from authors about some types of materials, experimental systems and methods used in many studies. Here, indicate whether each material, system or method listed is relevant to your study. If you are not sure if a list item applies to your research, read the appropriate section before selecting a response.

Materials & experimental systems

n/a	Involved in the study
☒	☐ Antibodies
☒	☐ Eukaryotic cell lines
☒	☐ Palaeontology and archaeology
☒	☐ Animals and other organisms
☒	☐ Clinical data
☒	☐ Dual use research of concern
☒	☐ Plants

Methods

n/a	Involved in the study
☒	☐ ChIP-seq
☒	☐ Flow cytometry
☒	☐ MRI-based neuroimaging

Plants

Seed stocks

Report on the source of all seed stocks or other plant material used. If applicable, state the seed stock centre and catalogue number. If plant specimens were collected from the field, describe the collection location, date and sampling procedures.

Novel plant genotypes

Describe the methods by which all novel plant genotypes were produced. This includes those generated by transgenic approaches, gene editing, chemical/radiation-based mutagenesis and hybridization. For transgenic lines, describe the transformation method, the number of independent lines analyzed and the generation upon which experiments were performed. For gene-edited lines, describe the editor used, the endogenous sequence targeted for editing, the targeting guide RNA sequence (if applicable) and how the editor was applied.

Authentication

Describe any authentication procedures for each seed stock used or novel genotype generated. Describe any experiments used to assess the effect of a mutation and, where applicable, how potential secondary effects (e.g. second site T-DNA insertions, mosiacism, off-target gene editing) were examined.
